# Glycosome turnover in *Leishmania major* is mediated by autophagy

**DOI:** 10.4161/auto.36438

**Published:** 2015-01-28

**Authors:** Benjamin Cull, Joseane Lima Prado Godinho, Juliany Cola Fernandes Rodrigues, Benjamin Frank, Uta Schurigt, Roderick AM Williams, Graham H Coombs, Jeremy C Mottram

**Affiliations:** 1 Wellcome Trust Center for Molecular Parasitology; Institute of Infection, Immunity and Inflammation; College of Medical, Veterinary and Life Sciences; University of Glasgow; Glasgow, UK; 2 Laboratório de Ultraestrutura Celular Hertha Meyer; Instituto de Biofísica Carlos Chagas Filho; Universidade Federal do Rio de Janeiro; Rio de Janeiro, Brazil; 3 Instituto Nacional de Ciência e Tecnologia de Biologia Estrutural e Bioimagem; Rio de Janeiro; Brazil; 4 Núcleo Multidisciplinar de Pesquisa em Biologia (NUMPEX-BIO); Polo Avançado de Xerém; Universidade Federal do Rio de Janeiro; Duque de Caxias, Brazil; 5 Instituto Nacional de Metrologia; Qualidade e Tecnologia; Inmetro; Rio de Janeiro, Brazil; 6 Institute for Molecular Infection Biology; University of Wuerzburg; Wuerzburg, Germany; 7 Strathclyde Institute of Pharmacy and Biomedical Sciences; University of Strathclyde; Glasgow, UK

**Keywords:** adaptation, ATG8, autophagy, glycosome, *Leishmania*, protozoan parasite, ATG, autophagy-related, GFP, green fluorescent protein, mC, mCherry fluorescent protein, MVT, multivesicular tubule, RFP, red fluorescent protein, TEM, transmission electron microscopy

## Abstract

Autophagy is a central process behind the cellular remodeling that occurs during differentiation of *Leishmania*, yet the cargo of the protozoan parasite's autophagosome is unknown. We have identified glycosomes, peroxisome-like organelles that uniquely compartmentalize glycolytic and other metabolic enzymes in *Leishmania* and other kinetoplastid parasitic protozoa, as autophagosome cargo. It has been proposed that the number of glycosomes and their content change during the *Leishmania* life cycle as a key adaptation to the different environments encountered. Quantification of RFP-SQL-labeled glycosomes showed that promastigotes of *L. major* possess ∼20 glycosomes per cell, whereas amastigotes contain ∼10. Glycosome numbers were significantly greater in promastigotes and amastigotes of autophagy-defective *L. major* Δ*atg5* mutants, implicating autophagy in glycosome homeostasis and providing a partial explanation for the previously observed growth and virulence defects of these mutants. Use of GFP-ATG8 to label autophagosomes showed glycosomes to be cargo in ∼15% of them; glycosome-containing autophagosomes were trafficked to the lysosome for degradation. The number of autophagosomes increased 10-fold during differentiation, yet the percentage of glycosome-containing autophagosomes remained constant. This indicates that increased turnover of glycosomes was due to an overall increase in autophagy, rather than an upregulation of autophagosomes containing this cargo. Mitophagy of the single mitochondrion was not observed in *L. major* during normal growth or differentiation; however, mitochondrial remnants resulting from stress-induced fragmentation colocalized with autophagosomes and lysosomes, indicating that autophagy is used to recycle these damaged organelles. These data show that autophagy in *Leishmania* has a central role not only in maintaining cellular homeostasis and recycling damaged organelles but crucially in the adaptation to environmental change through the turnover of glycosomes.

## Introduction

*Leishmania* are protozoan parasites responsible for the leishmaniases, diseases with clinical outcomes ranging from self-healing skin lesions to life-threatening infections of the liver, affecting millions of patients worldwide, primarily in tropical and subtropical regions.^1^ Within its sandfly insect vector, *Leishmania* replicate as motile flagellated procyclic promastigotes which differentiate, via several intermediate forms, into the infective metacyclic promastigotes that await transmission in the sandfly anterior midgut and mouthparts. Upon blood feeding, metacyclic promastigotes are released into the host and eventually make their way into macrophages, inside which the parasites differentiate into ovoid nonmotile amastigotes. Amastigotes reside and replicate within a lysosome-like parasitophorous vacuole inside host macrophages, evading and modulating the host immune system.^2^ *Leishmania* undergoes remodeling of its cellular architecture and metabolism to adapt to the different environments encountered in the insect vector and mammalian hosts.^3^ These remodeling events involve increased protein turnover, as evidenced by the evolution of lysosome morphology and associated increases in expression of peptidases.^3^ The lysosomal compartment occurs as a single large vesicular structure at the anterior end of procyclic promastigotes,^4,5^ whereas in the metacyclic promastigote it is tubular and known as the multivesicular tubule (MVT) or MVT-lysosome.^5,6^ Intracellular amastigotes possess characteristic large lysosomal compartments known as megasomes, which vary in size and number depending on the *Leishmania* species.^7,8^ These changes in lysosome morphology are reflected in increased expression of lysosomal cysteine peptidases in amastigotes^9,10^ and, similarly, an increase in overall proteolytic activity during procyclic to metacyclic promastigote differentiation.^6^

An important mechanism for protein turnover is autophagy, a conserved eukaryotic intracellular pathway by which cells target their own constituents to the lysosome for degradation and recycling. There have been 3 main types of autophagy described in mammalian cells. Chaperone-mediated autophagy is a process whereby cytosolic proteins possessing a KFERQ motif are transported into the lysosome for degradation with the aid of chaperones in a LAMP2A-dependent manner.^11^ Microautophagy involves invagination of the lysosomal membrane in order to directly engulf cytosol and organelles.^12^ However, the most studied type is macroautophagy, which is often referred to simply as ‘autophagy’ (as we shall do in this paper) and is characterized by the formation of a double-membrane-bound vesicle named the autophagosome. The autophagosome surrounds and sequesters regions of the cytosol containing proteins and organelles, before trafficking along microtubules to fuse with the lysosome where the contents are broken down by lysosomal enzymes. Before fusion with the lysosome, the autophagosome may also undergo fusion events with endosomal compartments. In addition to bulk degradation of cytosol, known as nonselective or bulk autophagy, selective types of autophagy have been described in higher eukaryotes where a specific cargo is sequestered for degradation. These include mitophagy, reticulophagy, pexophagy, nucleophagy, ribophagy, aggrephagy, and xenophagy, which describe the selective degradation of mitochondria, the endoplasmic reticulum (ER), peroxisomes, the nucleus, ribosomes, protein aggregates, and intracellular pathogens, respectively.^13-18^

The autophagy pathway is coordinated by ATG (autophagy-related) proteins, including the ubiquitin-like protein ATG8, which mediates expansion and completion of the autophagosome. As ATG8 remains associated with the autophagosome, GFP-ATG8 is a common molecular marker for tracking autophagosomes by fluorescence microscopy, from their formation to their delivery to the lysosome. Genes encoding proteins with similarity to the core ATG proteins in yeast and mammals have been described in *Leishmania* species,^19,20^ suggesting that these parasites possess a functional autophagy pathway. Further characterization showed that *Leishmania* expressing GFP-ATG8 form GFP-labeled puncta corresponding to autophagosomes and that autophagy is induced by starvation, as in yeast and mammals, as well as increasing during differentiation.^20,21^ During procyclic to metacyclic promastigote differentiation, GFP-ATG8 appears in tubular structures resembling the MVT-lysosome and the PE-conjugated form of ATG8 is more abundant as shown by western blotting. *Leishmania* with defects in the autophagy pathway, either overexpressing a dominant-negative VPS4, important in endosomal sorting pathways,^21^ or lacking the major lysosomal peptidases CPA and CPB,^20^ are unable to fully differentiate, in addition to being unable to survive prolonged starvation. These data show that autophagy is important for successful parasite differentiation, presumably because it is required for protein turnover during the cellular remodeling that occurs throughout the differentiation process. More recently, a *L. major* mutant lacking the gene encoding ATG5 (Δ*atg5*) has been characterized as unable to form autophagosomes, and as a consequence displays defects in differentiation, both during procyclic to metacyclic promastigote transformation and during differentiation from promastigotes to amastigotes in infected macrophages, as well as exhibiting mitochondrial abnormalities.^22^

Although it has been established that *Leishmania* undertake autophagy, the cargoes of autophagosomes have not been determined. We wished to examine which organelles might be targeted by autophagy during *Leishmania* differentiation as a mechanism of remodeling the organelles to adapt the parasite to its new life cycle environment. Kinetoplastid parasites, such as *Leishmania* and trypanosomes, possess unique organelles that are specialized to the lifestyles of these organisms. These include the glycosomes, peroxisome-related microbodies that contain enzymes of several important metabolic pathways including glycolysis, the pentose-phosphate pathway, β-oxidation of fatty acids, gluconeogenesis, purine salvage, and biosynthesis of pyrimidines, ether lipids and squalenes.^23^ Compartmentalization of metabolic pathways in this manner prevents the accumulation of toxic intermediates which would otherwise damage the cell,^24^ and is also thought to facilitate rapid metabolic adaptation in response to changing environments.^23^ Studies in both *Leishmania* and trypanosomes have shown that proper biogenesis of glycosomes and the correct targeting of glycosomal enzymes are essential for parasite survival,^24-29^ making these unique organelles an attractive drug target. An increased association of glycosomal markers with the lysosome during the differentiation of *Trypanosoma brucei*, from the short-stumpy trypomastigote to the procyclic trypomastigote, has been reported and this was interpreted by the authors as microautophagy and thought to be important for adaptation of the parasite's metabolic machinery in readiness for the next life-cycle environment.^30^ Turnover of glycosomes has also been proposed to occur during differentiation of *Leishmania* from the extracellular promastigote stage in the sand fly to the intracellular amastigote residing within mammalian macrophages,^23^ although supporting data are lacking. Moreover, several studies have shown that glycolytic enzymes are upregulated in promastigotes, whereas in amastigotes enzymes required for gluconeogenesis and β-oxidation of fatty acids are more important.^31-33^ In this study we have tested the hypothesis that this turnover of glycosomes involves autophagy. Pexophagy, the turnover of peroxisomes via autophagy, is a well-described phenomenon in methylotrophic yeasts, and can be induced by changing the nutrient source from methanol to glucose or ethanol. This results in peroxisomes that contain enzymes required for metabolism of methanol being degraded by autophagy, and new ones containing enzymes for metabolism of the new energy source are synthesized.^34^ Another interesting aspect of *Leishmania* biology is the presence of a single mitochondrion, which extends throughout the cell as a large reticulated network. In contrast, yeast and mammalian cells possess multiple mitochondria and those that are redundant or malfunctioning are removed by mitophagy. It was unclear how the necessary maintenance of the single mitochondrion in *Leishmania* can be achieved or whether autophagy is involved, and so we also aimed to address this question in this study.

## Results

### Assessment of glycosome numbers in L. major and in an autophagy-deficient mutant

**Figure 1 (See previous page).**
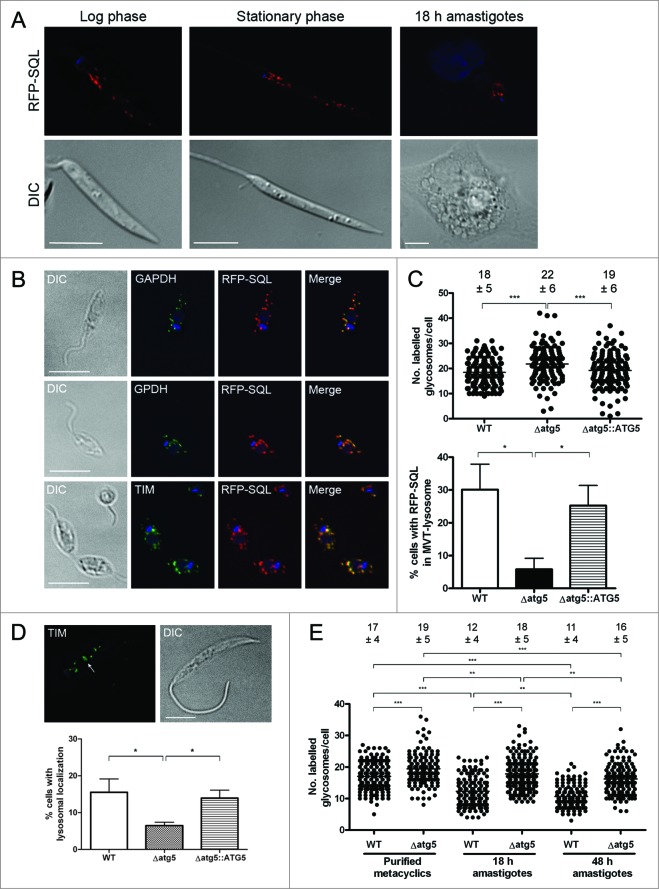
Comparison of glycosome numbers in WT and Δ*atg5* mutants. (**A**) *L. major* expressing RFP-SQL were imaged by fluorescence microscopy. Scale bar = 5 μm. (**B**) *L. major* expressing RFP-SQL were fixed and permeabilized, then probed with primary antibodies to various glycosomal enzymes. After addition of Alexa Fluor 488-conjugated secondary antibody, cells were mounted in VectaShield with DAPI and imaged by fluorescence microscopy. Scale bar = 10 μm. (**C**) Top: The number of glycosomes per cell was counted for at least 150 stationary phase promastigotes for each cell line. Below: The percentage of cells with MVT-lysosomes containing RFP-SQL. (**D**) Top: Immunofluorescence analysis of *L. major* promastigotes using anti-TIM antibodies. Arrow shows localization of anti-TIM to MVT-lysosome. Scale bar = 5 μm. Below: The percentage of cells containing MVT-lysosomes labeled by anti-TIM antibody. (**E**) Purified metacyclic promastigotes were used to infect murine peritoneal macrophages. Glycosomes per cell were counted in metacyclic promastigotes, and in parasites within macrophages either 18 or 48 h after infection. The number of glycosomes per cell was counted for at least 150 cells for each cell line at each time point. On scatter plots, each spot represents a single measurement with the horizontal line representing the mean. The mean ± standard deviation is included above each dataset. On bar charts, bars show mean of 3 independent experiments with error bars showing standard deviation. All data were analyzed using a one-way ANOVA with a Tukey-Kramer multiple comparison test. *** denotes *P* < 0.001, ** *P* < 0.01 and * *P* < 0.05.

To investigate glycosomes in *L. major,* procyclic promastigotes that constitutively expressed RFP-SQL were generated. The C-terminal SQL motif is a PTS1-type glycosomal targeting sequence^26,35^ and allows visualization of RFP-labeled glycosomes by live cell fluorescence microscopy in different life cycle stages (**Fig. 1A**). The exclusive localization of RFP-SQL to glycosomes was confirmed by colocalization studies with antibodies against known glycosomal enzymes GAPDH (glyceraldehyde-3-phosphate dehydrogenase), TIM (triosephosphate isomerase) and GPDH (glycerol-3-phosphate dehydrogenase) (**Fig. 1B**).^36^ We analyzed glycosome turnover by autophagy through the study of glycosome numbers in wild-type (WT) *L. major* and Δ*atg5* mutants which are unable to form autophagosomes and therefore are deficient in autophagy.^22^ Comparison of abundance of RFP-SQL-labeled glycosomes in stationary phase promastigotes of WT, Δ*atg5* and Δ*atg5::ATG5* (a line re-expressing ATG5 in the Δ*atg5* null mutant),^22^ revealed that the Δ*atg5* parasites contained higher numbers (average of 22 +/− 6 glycosomes/cell) compared with both WT (average of 18 +/− 5 glycosomes/cell) and Δ*atg5::ATG5* (average of 19 +/− 6 glycosomes/cell) (**Fig. 1C****, Video S1**). This small, but significant (*P* < 0.001), difference suggests that the lack of autophagy resulted in a decreased turnover of glycosomes in the Δ*atg5* promastigotes. In support of this, Δ*atg5* parasites also had significantly (*P* < 0.05) fewer RFP-SQL-containing MVT*-*lysosome-like structures when compared to WT and Δ*atg5::ATG5* parasites (**Fig. 1C**). The association of glycosomal enzymes to the MVT-lysosome (see also **Fig. 4 and 5** for MVT-lysosome characterization) was also reduced in Δ*atg5* parasites compared to WT and Δ*atg5::ATG5* (**Fig. 1D**). We found that glycosome numbers did not differ significantly between log phase and stationary phase WT promastigotes (**Fig. S1**), suggesting that glycosome abundance does not vary greatly between procyclic and metacyclic promastigotes. As a greater reduction in glycosome numbers occurs during metacyclic promastigote to amastigote differentiation, the numbers of RFP-SQL-labeled glycosomes were next determined for WT and Δ*atg5* parasites before, during and after parasite differentiation within macrophages. Glycosome numbers in purified metacyclic promastigotes were slightly, but significantly, higher in Δ*atg5* parasites (average 19 +/− 5 glycosomes/cell) compared to those in WT (average 17 +/− 4 glycosomes/cell; **Fig. 1E**). 18 h following infection of murine macrophages, the glycosome numbers in Δ*atg5* remained at levels similar to those in promastigotes (average 18 +/− 5 glycosomes/cell), whereas in WT parasites, numbers had decreased to an average 12 +/− 4 glycosomes/cell (**Fig. 1E**). 48 h following macrophage infection, by which time intracellular parasites should have differentiated into the amastigote form, glycosome numbers in Δ*atg5* had dropped slightly to an average 16 +/− 5 glycosomes/cell, but still remained significantly higher than those of WT parasites (average 11 +/− 4 glycosomes/cell) (**Fig. 1E**). These observations support the hypothesis that glycosomes are degraded by autophagy, and that this is important for the regulation of glycosome numbers during differentiation.

### Analysis of glycosome turnover by autophagy

**Figure 2.**
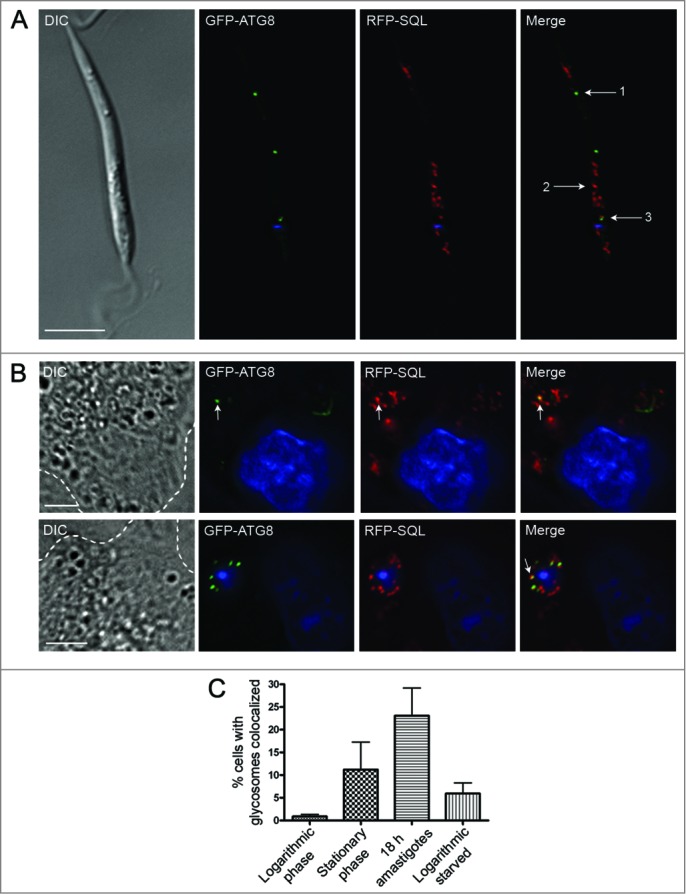
Analysis of glycosomes colocalized with autophagosomes. (**A**) *L. major* promastigotes coexpressing GFP-ATG8 (to label autophagosomes green) and RFP-SQL (to label glycosomes red) were DAPI-stained, washed and resuspended in PBS before live-cell imaging. The Pearson coefficient (*r*) represents the degree of colocalization between GFP-ATG8 and RFP-SQL. Arrows show examples of analysis of various structures: 1. GFP-ATG8 labeled autophagosome, *r* = 0.08. 2. RFP-SQL-labeled glycosome, *r* = 0.28. 3. GFP-ATG8 and RFP-SQL colocalization, *r* = 0.69. Scale bar = 5 μm. (**B**) Purified *L. major* metacyclic promastigotes coexpressing GFP-ATG8 and RFP-SQL were used to infect murine peritoneal macrophages. 18 h after infection, cells were stained with DAPI before live-cell imaging. Arrows show colocalization (*r* = 0.77 and *r* = 0.73 in upper and lower panels respectively). Images show a single slice from a 3-μm Z stack. Scale bar = 5 μm. Dashed lines show edges of macrophages. (**C**) Quantification of autophagosome-glycosome colocalization through the *L major* life cycle. The percentage of cells containing at least one GFP-ATG8-labeled autophagosome, and autophagosome-glycosome colocalization data (**Table S2**) were used to calculate the percentage of the population in which autophagosomes colocalized with glycosomes at any given time. Bars represent the mean and error bars the standard deviation of data from multiple experiments. A summary of these data can be found in **Table S1**.

**Figure 3.**
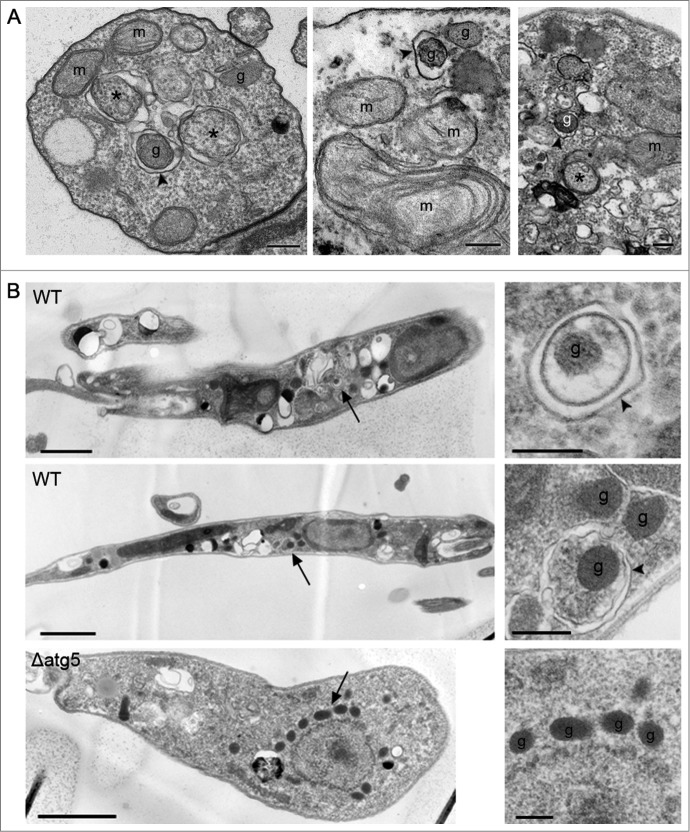
Visualization of glycosome turnover by transmission electron microscopy. (**A**) Ultrathin sections of *L. major* promastigotes in stationary phase (left panel) or from log phase and starved in PBS (middle and right panels). Scale bars = 0.2 μm. Arrowheads mark double-membrane vesicle-forming autophagosomes; asterisks mark cytosol within vesicle; g, glycosome; m, mitochondrion. (**B**) *L. major* WT promastigotes were starved for 4 h in PBS and *L. major* Δ*atg5* were grown in normal medium prior to preparation for TEM. Arrows in left panels show the structures enlarged in the adjacent panels. In enlarged images: arrowheads label autophagosomes; g, glycosome.

To investigate further the role of autophagy in glycosome turnover, parasites with RFP-labeled glycosomes were transfected with a construct that expresses the autophagosome marker GFP-ATG8.^20,21^ GFP-labeled autophagosomes were observed colocalizing with RFP-SQL-labeled glycosomes during promastigote log phase, stationary phase, and starvation (**Fig. 2A****; Table S1**), suggesting that glycosomes constitute autophagosome cargo in *Leishmania.* To measure the extent to which autophagosomes and glycosomes colocalized, and to test whether there is a turnover of glycosomes during differentiation, a detailed analysis of these interactions was performed. Colocalization events between GFP-ATG8 and RFP-SQL were analyzed by the Pearson Correlation Coefficient (**Fig. 2** **and Fig. S2**), with a value of 0.5 or above used as the criterion for autophagosome-glycosome colocalization (**Fig. 2A**, arrow 3). Glycosomes not colocalizing with autophagosomes (**Fig 2A**, arrow 2) and autophagosomes not colocalizing with glycosomes (**Fig 2A**, arrow 1) were also identified. The same method was used to assess autophagosome-glycosome colocalization in differentiating amastigotes within murine macrophages (**Fig. 2B**), in which autophagosomes were also observed to colocalize with glycosomes. As previously characterized in *L. major*,^21^ the percentage of promastigotes with autophagosomes was 4% cells in normal log phase growth and this increased upon starvation to 19%, and to 42% during stationary phase (**Tables S1 and S2**). In procyclic promastigotes, the percentage of autophagosomes colocalizing with glycosomes was approximately 16 to 19% whether in log phase, stationary phase, or after 4 h starvation of log phase cells in PBS (**Tables S1 and S2**). The increased autophagic induction during the stationary phase and starvation leads to a corresponding increase in glycosome turnover proportional to the increase in autophagy (**Fig. 2C**) due to the fact that at any time 16 to 19% of autophagosomes contain glycosomes as their cargo. Autophagosomes similarly colocalized with glycosomes during differentiation of metacyclic promastigotes to amastigotes within murine macrophages (**Fig. 2B** **and Fig. S2**); this analysis was performed 18 h following infection, as this is the time period at which autophagosome numbers are greatest during metacyclic promastigote to amastigote transformation in macrophages.^20^ At this stage of the life cycle, 90% of parasites contained autophagosomes; 11% of these colocalized with glycosomes (**Tables S1 and S2**). This was the stage when the greatest percentage of cells with autophagosome-glycosome colocalization was observed (**Fig. 2C**). These data show that glycosomes appear to be cargo for a subset of autophagosomes throughout the parasite's life cycle. The percentage of autophagosomes colocalized with glycosomes appears to remain relatively constant, but autophagy itself is upregulated in different life-cycle stages, which would therefore result in an increase in glycosome turnover. This conclusion is supported by the observation that in stationary phase promastigotes and during metacyclic promastigote to amastigote differentiation approximately 3% of glycosomes were found to be colocalized with autophagosomes, compared with only 1% in log phase promastigotes (**Table S3**). In order to confirm the presence of glycosome-containing autophagosomes and other structures related to autophagy, *L. major* promastigotes in log phase under PBS starvation, or in stationary phase, were analyzed by transmission electron microscopy (TEM). Glycosomes, identified on the basis of size and electron density, were detected within double-membrane-bound autophagosome structures (**Fig. 3**). In addition, membranes could be seen apparently forming around single glycosomes, as well as vacuoles containing parts of the cytoplasm (**Fig. 3A**). These structures were not observed in Δ*atg5* promastigotes, although increased numbers of glycosomes were apparent in these cells (**Fig. 3B**, lower panel); this is consistent with the absence of autophagy in these mutants.

### Trafficking of autophagosomes and glycosomes to the lysosomal compartment

**Figure 4.**
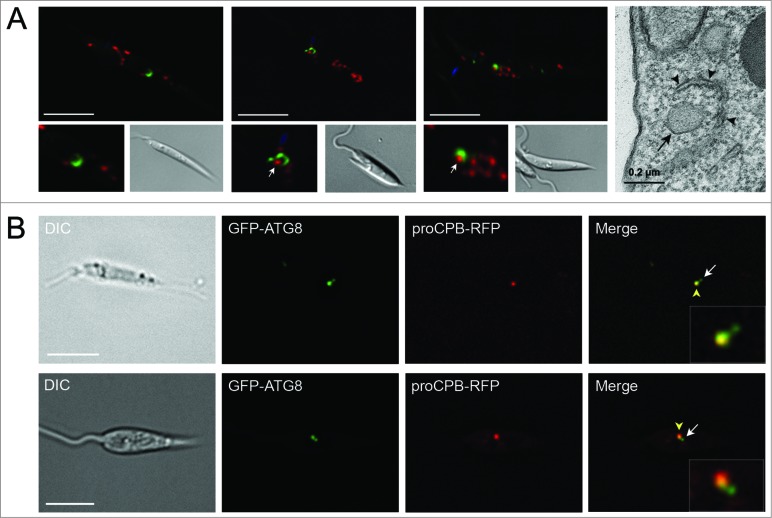
Visualization of autophagosome formation and fusion with lysosomes. (**A**) *L. major* promastigotes coexpressing GFP-ATG8 and RFP-SQL were imaged by fluorescence microscopy. Panels at lower left show enlarged images of cup-shaped autophagosomes. Panel on far right shows a section of a TEM of a stationary phase parasite. Arrows mark glycosomes that are being surrounded by autophagosomes. Arrowheads in TEM image show double-membrane forming around glycosome (marked with arrow). (**B**) Stationary phase *L. major* coexpressing GFP-ATG8 and proCPB-RFP. Enlarged images of autophagosome-lysosome fusion are displayed in insets. White arrow indicates autophagosome; yellow arrowhead marks lysosome containing GFP-ATG8 and proCPB-RFP. Images show a single slice from a 3-μm Z-stack. Scale bar = 5 μm.

**Figure 5.**
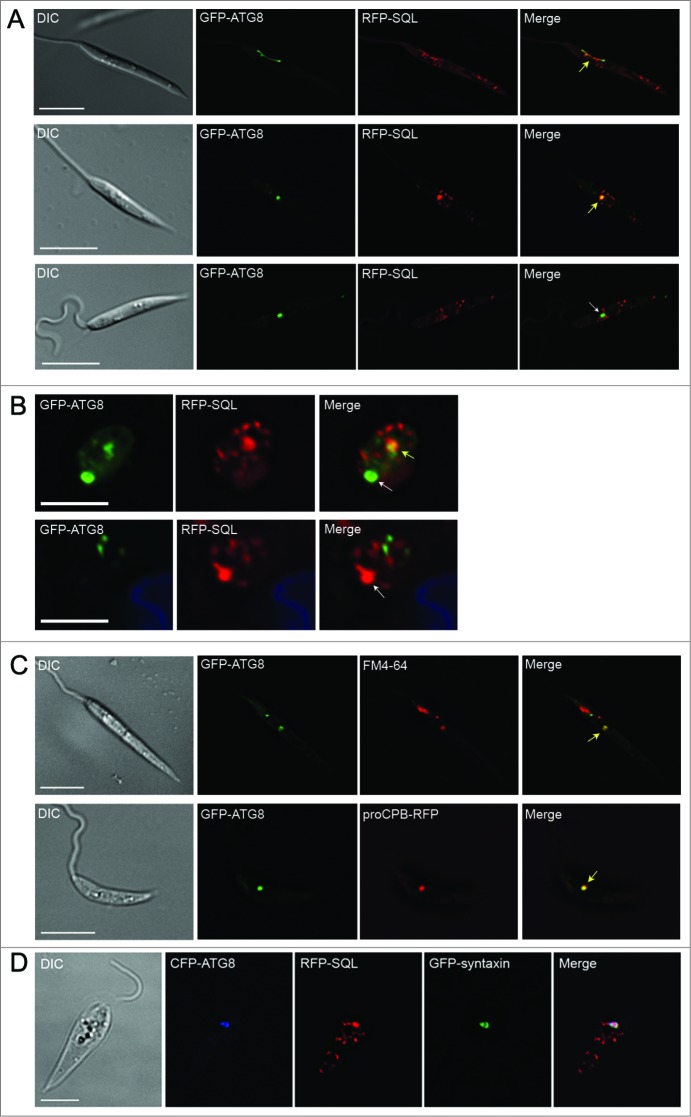
Trafficking of GFP-ATG8 to the lysosomal compartment. *L. major* promastigotes (**A**) or amastigotes in murine macrophages (**B**) coexpressing GFP-ATG8 and RFP-SQL were imaged by fluorescence microscopy. (**C**) Upper panel: stationary phase *L. major* promastigotes expressing GFP-ATG8 were incubated with 40 μM FM 4-64 for 1 h to label the lysosome for imaging. Lower panel: stationary phase *L. major* coexpressing GFP-ATG8 and proCPB-RFP were imaged by fluorescence microscopy. Yellow arrows show colocalization of signals in lysosomes; white arrows show localization of only GFP-ATG8 or RFP-SQL in lysosomes. (**D**) *L. major* promastigotes expressing CFP-ATG8, RFP-SQL and GFP-syntaxin. Images show a single slice from a 3-μm Z-stack. Scale bar = 5 μm.

In order to confirm that glycosomes are degraded by autophagy, rather than colocalization signifying some other autophagosome-glycosome interaction, autophagosome flux from formation to degradation was investigated. In addition to observation of GFP-ATG8 colocalizing with glycosomes, GFP-ATG8 was occasionally seen to label cup-shaped structures (**Fig. 4A**) resembling autophagosomes forming around single glycosomes. Similar structures, showing a double-membrane profile, were observed in proximity to glycosomes in stationary phase promastigotes examined by TEM (**Fig. 4A**). Furthermore, both GFP-ATG8 and RFP-SQL labeled large tubular structures in stationary phase promastigotes that resemble the MVT-lysosome, the lysosomal compartment of metacyclic promastigotes (**Fig. 5A**).^6^ In log phase parasites, as well as in some stationary phase parasites, GFP-ATG8 and RFP-SQL accumulated in large structures that we hypothesized might be lysosomes (**Fig. 5A**). These large structures were labeled with either GFP-ATG8 or RFP-SQL alone (**Fig. 5A**), and were visible during metacyclic promastigote-amastigote differentiation (**Fig. 5B**), corresponding to the megasomes of *Leishmania* amastigotes. To determine whether this compartment was the lysosome, we employed 2 different markers: FM 4–64^6,21^ and proCPB-RFP, the propeptide region of the lysosomal cysteine peptidase B.^4^ Both of these markers colocalized with GFP-ATG8 in large structures similar to those colabeled by GFP-ATG8 and RFP-SQL (**Fig. 5C**), showing that this compartment is the lysosome. Furthermore, colocalization of CFP-ATG8, RFP-SQL and the lysosomal SNARE GFP-syntaxin in this compartment confirmed the transport of autophagosomes and glycosomes to the lysosome (**Fig. 5D**). Additionally, smaller GFP-ATG8-labeled puncta were associated with large structures containing both GFP-ATG8 and proCPB-RFP, suggesting that the smaller puncta were autophagosomes in the process of fusing with lysosomes (**Fig. 4B**). Together these data show that GFP-ATG8 and RFP-SQL interact at all stages of the autophagy pathway and support the hypothesis that glycosomes are sequestered by autophagosomes for their subsequent trafficking to and degradation in the lysosome.

### Characterization of autophagosomes and lysosomes based on size and labeling

**Figure 6.**
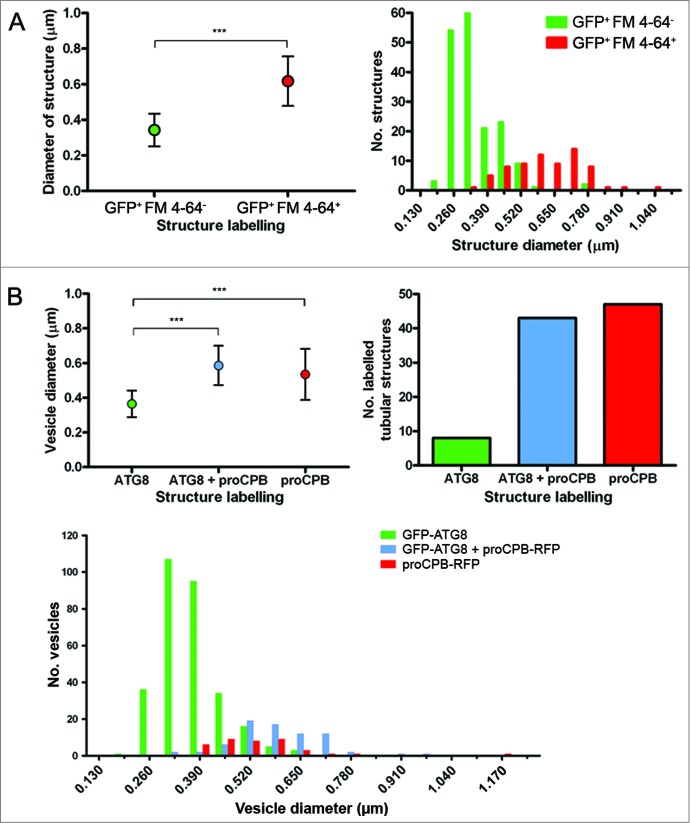
Characterization of vesicle size in *L. major* during stationary phase. (**A**) *L. major* expressing GFP-ATG8 were incubated with 40 μM FM 4–64 for 1 h to stain the lysosomal compartment, before imaging. Left: Average vesicle size. Dots represent the mean and the error bars standard deviation. Right: Frequency distribution of vesicle sizes. (**B**) *L. major* coexpressing GFP-ATG8 and proCPB-RFP. Left: Average vesicle size. Dots represent the mean and the error bars standard deviation. Right: Numbers of tubular compartments labeled with GFP-ATG8, proCPB-RFP or both. Bottom: Frequency distribution of vesicle sizes. Vesicle sizes were measured using SoftWoRx Explorer Image analysis software. Data were analyzed using the Student *t* test (for 2 samples; **A**) or one-way ANOVA with a Tukey-Kramer multiple comparison test (for 3 samples; **B**). Frequency distribution data were binned by 0.065 μm intervals, which correspond to the limit of measurement accuracy based on the microscopy image pixels. *** denotes *P* < 0.001.

To extend our analysis of the lysosomal compartment and to distinguish lysosomes from autophagosomes, a size comparison of labeled vesicles was carried out in stationary phase promastigotes expressing GFP-ATG8 and incubated with FM 4-64 for 1 h to allow it to accumulate in lysosomes. Measurements of vesicle diameter show that GFP-ATG8^+^ FM 4-64^−^ vesicles (autophagosomes) were on average significantly smaller (0.34 μm) than GFP-ATG8^+^ FM 4-64^+^ vesicles (autolysosomes/lysosomes, 0.62 μm; **Fig. 6A**). Frequency distribution of the data shows that the majority of GFP-ATG8^+^ FM 4-64^−^ vesicles were approximately 0.3 μm in diameter, whereas most GFP-ATG8^+^ FM 4-64^+^ vesicles were greater than 0.4 μm (**Fig. 6A**). Some overlap of GFP-ATG8^+^ FM 4-64^−^ vesicles with GFP-ATG8^+^ FM 4-64^+^ vesicles of size 0.4 – 0.6 μm suggests that this population may represent autolysosomes or a possible endosomal-autophagosomal intermediate, as autophagosomes interact with the endosomal system in mammalian cells.^37^ As FM 4-64 is not a specific lysosomal marker and may also be labeling endosomal compartments, it cannot be discounted that the large GFP-ATG8^+^ FM 4-64^+^ vesicles are large endosomal-autophagosomal fusions rather than lysosomes. Therefore a similar analysis of vesicle size was performed in stationary phase promastigotes expressing GFP-ATG8 and proCPB-RFP, which is known to label the lysosome.^4^ This more extensive analysis produced similar results, with GFP-ATG8-labeled vesicles exhibiting a mean diameter of 0.36 μm, whereas vesicles labeled with both GFP-ATG8 and proCPB-RFP, or proCPB-RFP alone, had mean diameters of 0.59 μm and 0.53 μm, respectively (**Fig. 6B**). The majority of structures labeled by GFP-ATG8 and proCPB-RFP together or proCPB-RFP alone were tubular compartments corresponding to the MVT-lysosome (**Fig. 6B**), which were not included in the analysis in **Fig. 6A**. Frequency distribution of vesicle size data corresponded to that seen with GFP-ATG8 and FM 4-64, with a population of smaller vesicles labeled with GFP-ATG8, that can be characterized as autophagosomes, a population of large vesicles that either displayed labeling with only proCPB-RFP or with both proCPB-RFP and GFP-ATG8, which are lysosomes, and a third population that occurred in the overlap between the other 2 populations, autolysosomes (**Fig. 6B**). This analysis has provided a classification of autophagosomes and lysosomes in *L. major* based on their size and labeling.

### Investigation of mitophagy during starvation

**Figure 7.**
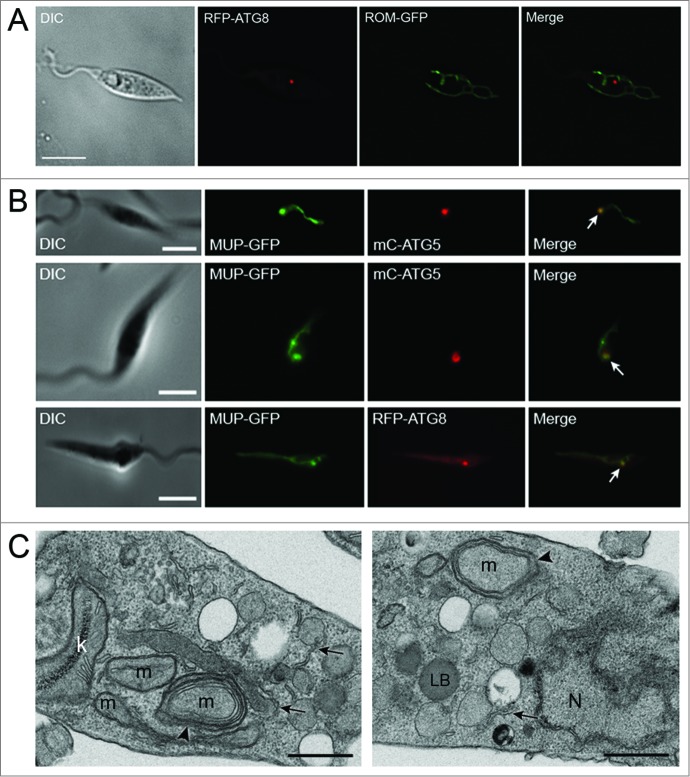
Analysis of autophagy and the mitochondrion during normal growth and starvation. (**A**) Stationary phase *L. major* promastigotes coexpressing RFP-ATG8 and ROM-GFP. (**B**) The occurrence and colocalization of mC-ATG5 or RFP-ATG8 puncta and MUP-GFP coexpressed in log phase WT *L. major* promastigotes after 4 h in PBS at 25°C. RFP-ATG8 or mC-ATG5 puncta colocalizing with MUP-GFP are arrowed. Images show a single slice from a 3-μm Z stack. Scale bar = 5 μm. (**C**) TEM. Ultrathin sections of *L. major* promastigotes after starvation in PBS. Arrowheads mark double-membrane vesicles formed around pieces of mitochondrion. Arrows mark ER profiles which may represent forming autophagic membranes. K, kinetoplast; LB, lipid body; m, mitochondrion; N, nucleus. Scale bars = 0.5 μm.

Mitochondrial quality control is crucial for the maintenance of cellular homeostasis in most cells but the mechanisms by which this occurs in *Leishmania* are not known.^22^ Our use of 2 mitochondrial markers, ROM-GFP, a marker for the inner mitochondrial membrane, and MUP-GFP, a marker for the outer mitochondrial membrane, expressed in promastigotes of *L. major* during normal growth provided no evidence for the occurrence of mitophagy (**Fig. 7A****, Table S4**).^22^ However, we decided to extend this search for mitophagy by including other growth conditions such as those known to induce mitophagy e.g. starvation.^38^ Log phase WT promastigotes coexpressing MUP-GFP and mC-ATG5 or RFP-ATG8 were starved for 4 h and analyzed by fluorescence microscopy. The reticulate network indicative of the single mitochondrion was observed under these conditions (**Fig. 7B**). However, there were regions along the network where the MUP-GFP accumulated and had a punctate appearance (**Fig. 7B**). These structures were present in approximately 47% of the starved promastigotes. As it seemed unlikely that the whole of the large unitary complex mitochondrial network is sequestered and digested via mitophagy, whereas portions of it may be, we examined if these punctate structures on the mitochondrial network colocalized with ATG5 or ATG8. Using WT promastigotes expressing mC-ATG5 or RFP-ATG8 and MUP-GFP ^22^ under the same conditions, 16% of cells had mC-ATG5 puncta, whereas 52% had RFP-ATG8 puncta (**Table S5**). MUP-GFP foci occurred in 46% of lines containing mC-ATG5 or RFP-ATG8. 62% of mC-ATG5 puncta colocalized with MUP-GFP foci (**Fig. 7B**, top and middle panels; **Table S5**) and only 27% MUP-GFP puncta were labeled with mC-ATG5 (**Table S5**). Similarly, 55% of RFP-ATG8 puncta colocalized with MUP-GFP puncta (**Fig. 7B**, bottom panel; **Table S5**) and 29% of MUP-GFP puncta were labeled with RFP-ATG8 (**Table S5**). However, under these conditions, neither mC-ATG5, RFP-ATG8 or MUP-GFP were observed in lysosomes; if sections of the mitochondrion were degraded by autophagy we would expect both cargo and autophagic proteins to be trafficked to the lysosome, as was seen during experiments with GFP-ATG8 and the glycosomal marker RFP-SQL (**Fig. 5**). Nor was MUP-GFP detected in mC-ATG5- or RFP-ATG8-labeled puncta that were not in contact with the mitochondrion, so it is unclear whether mitochondrial protein is trafficked to the lysosome by the classical autophagy pathway or whether the MUP-ATG5 or -ATG8 foci may represent another process. However, when starved promastigotes were viewed by TEM, sections of the mitochondrion appeared to be surrounded by double-membrane profiles in some images (**Fig. 7C**). This might suggest selective autophagic engulfment of some pieces of this organelle during starvation, as adjacent parts of the mitochondrion were not being sequestered by membranes in the same way (**Fig. 7C**, left panel). These images also show many ER profiles (**Fig. 7C**, arrowed), which may be related to autophagosome formation.

**Figure 8.**
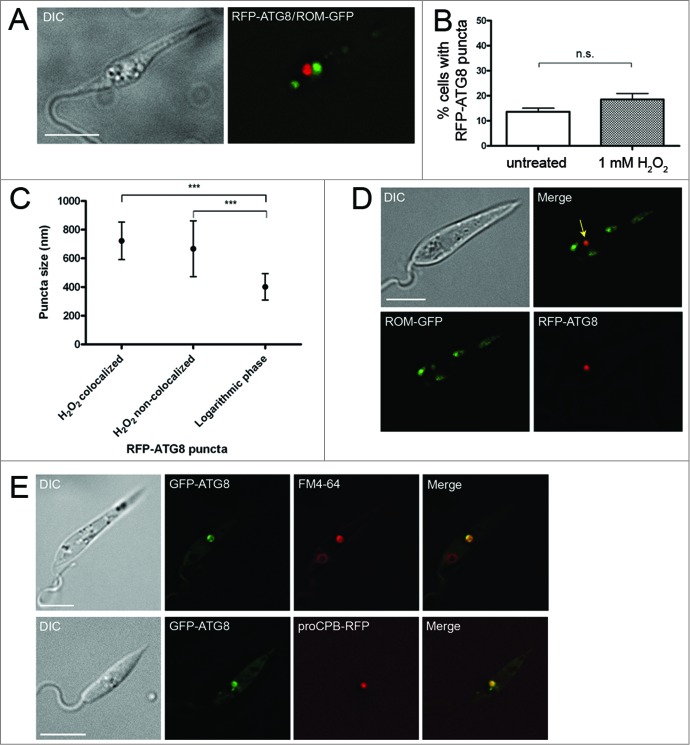
Analysis of autophagy during H_2_O_2_-induced mitochondrial fragmentation. (**A**) Log phase *L. major* coexpressing RFP-ATG8 and ROM-GFP were incubated with 1 mM H_2_O_2_ for 1 h at 25°C. (**B**) Percentage of cells exhibiting one or more RFP-ATG8 puncta was determined by counting 200 treated and 200 untreated cells per experiment. Bars show mean and error bars standard deviation of 4 independent experiments. Data were analyzed using a Student *t* test; n.s. not significant. (**C**) Puncta sizes were measured using SoftWoRx Explorer Image Analysis software. Data were analyzed using one-way ANOVA with a Tukey-Kramer multiple comparison test. *** signifies *P* < 0.001. (**D**) Parasites were prepared as in A. Colocalization analysis was performed using SoftWoRx Image Analysis software. Arrow marks colocalization. (**E**) *L. major* expressing GFP-ATG8 were incubated with 40 μM FM 4-64 for 1 h before H_2_O_2_ treatment as above. Microscopy images show a single slice from a 3-μm Z-stack. Scale bar = 5 μm.

The data suggest that autophagic degradation of the mitochondrion may occur when the organelle is damaged. We hypothesized that inducing fragmentation of the mitochondrion would cause autophagy to sequester and degrade mitochondrial fragments. We used H_2_O_2_ to induce mitochondrial fragmentation in log phase promastigotes (as reported in ^39^). This resulted in fragmentation of the mitochondrion in the majority of cells (**Fig. 8A**) but did not significantly increase appearance of RFP-ATG8 puncta compared to an untreated control (**Fig. 8B**). However, RFP-ATG8 puncta were significantly larger in H_2_O_2_-treated cells (**Fig. 8A, C**). A small proportion (6.5%) of these vesicles was seen colocalizing with fragments of the mitochondrion labeled with ROM-GFP (**Fig. 8D**). Analysis of sizes of RFP-ATG8 puncta demonstrated that there was no difference in vesicle size between those that colocalized with mitochondrial fragments and those that did not (**Fig. 8C**). These data are consistent with mitophagy being responsible for some degradation of mitochondrial fragments under conditions where the mitochondrion is damaged and fragmented.

To determine whether the larger RFP-ATG8 puncta were due to increased trafficking of ATG8 to lysosomes, parasites expressing GFP-ATG8 were incubated with FM 4-64 for 1 h to label the lysosome before incubation in H_2_O_2_. All large GFP-ATG8 structures were seen to colocalize with FM 4-64 (**Fig. 8E**), suggesting that this compartment was lysosomal. To further examine the nature of these structures, parasites coexpressing GFP-ATG8 and the lysosome-specific marker, proCPB-RFP, were examined after H_2_O_2_ treatment, and in these parasites large ATG8-labeled vesicles also contained proCPB-RFP (**Fig. 8E**) identifying these structures as lysosomes.

### Other autophagosome cargo in L. major

Another possible organellar cargo in *Leishmania* that we considered is the acidocalcisome, which is important for osmoregulation and cation and pH homeostasis.^40^ It is conceivable that acidocalcisome numbers could be regulated to adapt the parasites to the changing conditions of different life cycle environments and that autophagy could be responsible for their degradation when fewer are needed. V-H^+^-PPase-GFP was used as a marker of acidocalcisomes.^41^ V-H^+^-PPase-GFP-labeled acidocalcisomes were detected, but no colocalization of these with RFP-ATG8 autophagosomes was evident, whether promastigotes were in log phase, stationary phase, or had been starved for 4 h (**Fig. S3; Table S6**). Neither was V-H^+^-PPase-GFP ever observed in lysosomal structures containing RFP-ATG8. These data suggest that acidocalcisomes are not significant cargo for autophagosomes, at least in promastigote life cycle stages.

## Discussion

An important aspect of autophagy in *Leishmania* is its role in cellular remodeling during differentiation between parasite life-cycle stages.^20,21^ In this study we have investigated which organelles constitute cargo for autophagy during differentiation and under normal or nutrient-deprived growth conditions, with emphasis on specialized organelles of this protozoan parasite and in particular the glycosome. It had been proposed that glycosome numbers decrease, and that changes in metabolic enzymes occur, during the transformation of promastigotes into amastigotes.^31-33^ This study provides the first quantification of glycosome numbers within the different life cycle stages of *L. major,* using 3D fluorescence microscopy. We found that the number of glycosomes in procyclic and metacyclic promastigotes was similar (being ∼20), but the number was reduced some 2-fold upon differentiation into the amastigote (**Fig. 1E**); the 10 detected in the latter form being comparable with previous estimates from serial EM sections of *L. mexicana* amastigotes.^7^ Glycosomes labeled with RFP-SQL were significantly higher in numbers in stationary phase Δ*atg5* autophagy-deficient parasites than in WT parasites in the same stage of growth (**Fig. 1C**), and decreased delivery of glycosomes to lysosomal structures was observed, consistent with the idea that autophagy is important for glycosome turnover during procyclic to metacyclic promastigote differentiation. The numbers support the hypothesis that some glycosomes are replaced with newly synthesized organelles that contain different configurations of metabolic enzymes to adapt parasites to infection of the mammalian host. The existence of autophagosome-glycosome colocalization and appearance of both GFP-ATG8 and RFP-SQL in lysosomes during normal growth also suggests that there is some constitutive maintenance of the glycosome population by autophagy. Increased glycosome turnover was also seen during starvation, which suggests that under such extreme conditions the organelles can be degraded to provide essential amino acids and other materials to facilitate cell survival.

The observation of cup-shaped structures that appeared to be forming around single glycosomes (**Fig. 4A**), the accumulation of both GFP-ATG8 and the glycosomal marker RFP-SQL within lysosomal compartments (**Fig. 5**), and the visualization of GFP-ATG8 puncta undergoing fusion with the lysosome (**Fig. 4B**) all support a turnover mechanism whereby single glycosomes are targeted by autophagosomes and trafficked to the lysosome for degradation. This suggests that an important mechanism of glycosome turnover in *Leishmania* is autophagy involving autophagosomes, a mechanism supported by our TEM analysis of *L. major* promastigotes (**Fig. 3**). TEM data for other kinetoplastids also support this hypothesis: double-membrane structures enclosing glycosomes can be observed in early micrographs of *Trypanosoma congolense*,^42^ and glycosomal proteins were detected by immuno-EM within the lysosome of *T. brucei* during differentiation.^30^ However, the observation that glycosome numbers do decrease in *L. major* Δ*atg5* parasites during differentiation from promastigote to amastigote, and that some RFP-SQL localizes to lysosomal compartments in stationary phase Δ*atg5* cells, indicates that glycosomes are also degraded by other mechanisms. One possibility is that microautophagy also functions and our finding that in some cells RFP-SQL-labeled glycosomes appeared to be adjacent to the MVT-lysosome (**Fig. 5A**, top panels), although not a frequent observation, could reflect this. Selective forms of microautophagy in yeast are, however, thought to require the core autophagy machinery, including ATG5,^43–45^ thus could not operate in the Δ*atg5* cells. Nonselective microautophagy has been reported not to require ATG5^46,47^ and so this pathway could occur in the Δ*atg5 Leishmania*. It is interesting that although glycosome turnover was reduced in Δ*atg5* parasites, the numbers of glycosomes in these cells were not markedly increased. A doubling in numbers could have occurred if a full number of glycosomes had been newly synthesized and yet all glycosome degradation ceased. This implies that other mechanisms of glycosome degradation occur and also that feedback mechanisms exist to maintain glycosome numbers at manageable levels in the absence of autophagy involving autophagosomes operating to remove redundant glycosomes. Microautophagy has been suggested as the main route for degradation of glycosomes in *T. brucei,*^30^ although this analysis used markers of glycosomes and the lysosome without specifically labeling autophagosomes and so it is possible that macroautophagy could also be involved in this parasite.

We found that throughout the life cycle of *L. major* ∼15% of autophagosomes colocalize with glycosomes even though autophagy is upregulated ∼10-fold during differentiation.^20,21^ This could result in up to a 10-fold increase in glycosome turnover during differentiation (**Fig. 2C**), and we observed an increase in the percentage of total glycosomes colocalizing with autophagosomes from 1% during log phase growth to 3% during differentiation (**Table S3**). Therefore, depending on the time it takes for an autophagosome to sequester and traffic its cargo to the lysosome (observed to be ∼16 min during mitophagy in mammals),^48^ it is possible that the whole population of glycosomes could be turned over by autophagy within ∼8 h; this is consistent with the timescale of differentiation. This would result in a gradual degradation of glycosomes while new organelles could be synthesized, a process which would require selective turnover of old vs. new glycosomes. Our results could be consistent with the presence of a specific set of autophagosomes that target glycosomes, supporting the idea that glycosomes are selective cargo. In starved rat hepatocytes, 85% of autophagosomes were observed to contain mitochondria^48^ and it was concluded that under these conditions these organelles were being nonselectively sequestered by the majority of autophagosomes. Our data for glycosomes in starving promastigotes suggest that they are not the major source of nutrients targeted via nonselective autophagy to ensure survival.

It is possible that DAPI, which binds tightly to DNA, may have some effect on autophagy, a pathway induced by DNA damage in mammals.^49^ However, previous work concludes that DAPI causes no significant mutagenic or genotoxic effects despite its DNA binding properties.^50^ We observed autophagosome formation during log phase, differentiation, and starvation occurs in DAPI-stained *L. major* at a similar rate to that reported in other studies in which parasite DNA is not stained.^21,51^ Therefore, DAPI treatment does not appear to exert any significant effect on the *Leishmania* autophagy pathway.

Analysis of the sizes of compartments differentially labeled by GFP-ATG8 and markers of the lysosome has provided new data on the vesicles involved in the autophagy pathway in *Leishmania* (**Fig. 6**). Autophagosomes under normal growth conditions had an average of 0.30 to 0.36 μm in diameter, making them smaller than those of yeast, which were measured by electron microscopy to have a diameter in the range 0.3 to 0.9 μm,^52,53^ and mammalian cells, which measure 0.5 to 1.0 μm.^48,54^ The autophagosomes of *L. major* were relatively uniform in size (**Fig. 6**), both during normal growth and starvation conditions, suggesting that their cargo normally was no larger than ∼0.3 μm wide. In contrast, under extreme stress such as H_2_O_2_ treatment, which caused mitochondrial fragmentation, larger GFP-ATG8-labeled autophagosome structures occurred (average diameter 0.6 to 0.7 μm), presumably reflecting the size and/or quantity of their cargo under these conditions. The single *Leishmania* lysosome had a mean diameter of 0.5 to 0.6 μm; in contrast, yeast typically contain 1 to 3 large vacuoles 1 to 5 μm in diameter^53^ and mammalian cells contain multiple small lysosomes 0.2 to 1.5 μm in size.^48^ These data provide a useful reference for recognition of *Leishmania* compartments based on their size and shape, as well as comparison with those of other organisms.

Autophagy plays an important role in mitochondrial maintenance in mammalian cells. Mitochondria are constantly undergoing fusion and fission, and it is thought that damaged mitochondria that are unable to fuse to the mitochondrial network are removed by autophagy.^55^ The process preferentially targets small mitochondria or mitochondrial fragments and, indeed, during nutrient starvation mitochondria fuse together to form an elongated network which protects them from autophagic degradation.^56,57^ These observations on mitophagy raise the question of how *Leishmania* and other organisms with a single mitochondrion maintain homeostasis of this organelle, discussed previously by Rigden and colleagues.^58^ We were unable to find evidence for mitophagy during normal growth of *L. major* (**Fig. 7A**),^22^ suggesting that mechanisms other than autophagy are involved in the organelle's maintenance. It is known that autophagy-independent pathways for the degradation of mitochondrial proteins exist in other organisms, especially under stress.^59,60^ Our findings suggest that autophagy may be responsible for clearing damaged regions or fragments of the parasite's single mitochondrion during stress: after treatment of promastigotes with 1 mM H_2_O_2_ a low number of GFP-ATG8-labeled puncta colocalized with mitochondrial fragments (**Fig. 8**). These puncta, however, were relatively large and colabeled with lysosomal markers, raising the possibility that this sequestration of mitochondrial fragments may occur by a process other than macroautophagy, perhaps through microautophagy. Starvation generally induces autophagy and in mammalian cells this results in an ordered degradation process. Cytosolic proteins are preferentially degraded during early starvation and proteins from large organelles, such as mitochondria, targeted only at later stages of starvation i.e., >24 h.^61^ This suggests that accessible energy sources are targeted first before degradation of organelles that are energetically costly to synthesize and important for survival. Our studies have shown that glycosomes are not preferentially targeted during starvation but they do suggest that prolonged starvation of *Leishmania* might induce autophagic turnover of the mitochondrion, with damaged parts of the unitary organelle being targeted (**Fig. 7B, C**). It is also possible that parts of the mitochondrion may be remodeled by autophagy during promastigote to amastigote differentiation, which was not examined in our study. The other organelle that was investigated here (acidocalcisomes) does not appear to constitute autophagosome cargo during starvation or procyclic to metacyclic differentiation, although it is possible that they could be degraded by autophagy at other stages of the life cycle.

The importance of autophagy pathways for the homeostasis, turnover and adaptation of glycosomes, and therefore *leishmania*'s metabolism, may partly explain the growth and virulence defects observed in *L. major* parasites lacking the gene encoding ATG5.^22^ *L. major* lacking ATG4.1 or ATG4.2, which have abnormal autophagy, also display low infectivity.^51^ ATG4 double mutants could not be generated,^51^ suggesting that the autophagy pathway and its molecular components intersect with other essential processes in *Leishmania*. Future studies on the autophagic pathways and how they help to adapt the parasites for infection and surviving stress will provide further insights into the mechanisms whereby cellular homeostasis is achieved in *Leishmania*.

## Materials and Methods

### Parasite culture

*Leishmania major* (MHOM/JL/80/Friedlin) were grown in modified Eagle's medium supplemented with 10% (v/v) heat-inactivated foetal calf serum (Gibco, 10500-064) at 25°C. When referring to the stage of growth of cultures, mid-log phase corresponds to ∼5 × 10^6^ parasites/ml and stationary phase to ∼2 × 10^7^ parasites/ml. Metacyclic promastigotes were isolated from late stationary phase cultures using peanut lectin agglutination.^62^ Transgenic parasites were maintained in appropriate antibiotics: G418 at 50 μg/ml; hygromycin B at 50 μg/ml; blasticidin S at 10 μg/ml; phleomycin at 10 μg/ml; puromycin at 50 μg/ml (InvivoGen, ant-gn; ant-hm; ant-bl; ant-ph; ant-pr). The generation of Δ*atg5* and Δ*atg5::ATG5* cell lines is described in detail in ref 22.

### Generation of cell lines expressing fluorescent markers

The generation of green fluorescent protein GFP-ATG8 and red fluorescent protein RFP-ATG8 plasmids are described in references^21^ and ^20^ respectively. Cerulean fluorescent protein CFP-ATG8 was produced by inserting the open reading frame (ORF) of CFP into the *Nde* I/*Bgl* II site of the GFP-ATG8 plasmid. The RFP-SQL glycosomal labeling construct was generated by inserting the ORF of RFP plus a C-terminal glycosomal targeting sequence (SQL) into the *Nde* I/*Xho* I site of the pNUS-RFPcD vector. Construction of the lysosomal markers proCPB-RFP and GFP-syntaxin has been described previously.^4,5^ The V-H^+^-PPase-GFP plasmid, used to label acidocalcisomes, was generated by inserting the ORF of LmjF31.1220 into the *Nde* I/*Kpn* I site of pNUS-GFPcN,^63^ as described in ref 41. The generation of ROM-GFP, MUP-GFP and mCherry-ATG5 plasmids is described in ref 22. Plasmids were transfected into *L. major* parasites using a Nucleofector transfection system (Lonza, AAB-1001).

### Induction and monitoring of autophagy

Autophagy was induced by starvation or differentiation as previously reported.^20,21^ Promastigotes were starved by washing 3 times in phosphate-buffered saline (PBS; 137 mM NaCl, 2.7 mM KCl, 10 mM phosphate buffer, pH 7.4), followed by resuspension in PBS at 1 × 10^8^ parasites/ml for up to 5 h. To assess autophagy during procyclic to metacyclic promastigote differentiation, cells were observed in stationary phase of growth;^21^ for metacyclic promastigote to amastigote differentiation, parasites were observed 18 h after infection of macrophages.^20^ Live parasites expressing GFP-ATG8 or RFP-ATG8 were observed by fluorescence microscopy (see below) and the presence and number of GFP- or RFP-ATG8-labeled autophagosomes within these cells were recorded. The level of autophagy is expressed as the percentage of cells in a population containing at least one GFP- or RFP-ATG8-labeled autophagosome; these measurements were made from counting at least 200 cells from at least 3 independent experiments.

### Live-cell imaging

For live-cell imaging, cells were pelleted by centrifugation then washed twice in PBS to remove medium, before resuspension in PBS and mounting live for microscopy. Parasites were viewed by fluorescence microscopy on a DeltaVision Core deconvolution microscope (Applied Precision Inc., Issaquah (WA), USA) with GFP, mCherry, CFP, and DAPI filters, and imaged with a Photometrics CoolSNAP HQ^2^ camera. Imaging of parasites was performed at 100 x magnification, and infected macrophages at 60 x, and was limited to <1 h per sample to maintain cell viability. DIC images were obtained under polarized light. Typically, images were taken through a 3-μm Z-stack with 0.2 μm between each image in the stack. Deconvolution was performed using a conservative ratio method with 10 iterations. Images were processed and analyzed using SoftWoRx image analysis software (Applied Precision Inc., Issaquah (WA), USA).

### Colocalization analysis

Colocalization analyses were performed using the Colocalization Finder tool of SoftWoRx image analysis software (Applied Precision Inc., Issaquah (WA), USA). This uses the Pearson correlation coefficient (*r*) to calculate the correlation between 2 variables; in this case the intensity of the 2 fluorophores within a selected area, with values close to zero indicating no colocalization and values close to one indicating that the fluorophores are located within the same area.^64^ When a GFP- or RFP-ATG8-labeled autophagosome was suspected to be colocalizing with a fluorescent organellar marker, the region of the image containing the autophagosome and cargo was selected and a colocalization analysis performed on this region. Due to the way autophagosomes and their cargo would be arranged in space during cargo turnover, i.e. with the engulfed cargo *inside* the autophagosome, rather than on the same structure, an *r* > 0.5 and above was used as the criterion for genuine colocalization.

### Immunofluorescence analysis

Promastigotes were washed twice in PBS, fixed in 1% formaldehyde for 30 min, then permeabilized by addition of 0.1% Triton X-100 in PBS for 10 min. Cells were resuspended in 0.1 M glycine in PBS for a further 10 min before being washed in PBS, and then added to poly-*L*-lysine-coated slides (Sigma-Aldrich, P4707) and allowed to dry. Following 5 min rehydration in TB buffer [0.1% (v/v) Triton X-100 (Sigma-Aldrich, T8787), 0.1% (w/v) bovine serum albumin (BSA; Sigma-Aldrich, A9418) in PBS], antibodies to *Leishmania* glycosomal antibodies (rabbit anti-GAPDH, anti-TIM and anti-GPDH, diluted 1:1000 in TB buffer), were added to slides and incubated for 1 h at room temperature. After washing slides in PBS, the secondary antibody (Alexa Fluor 488-conjugated goat anti-rabbit; Molecular Probes, A-11008) was added to slides at 1:500 in TB buffer and incubated for 1 h in the dark. Finally, cells were mounted in VectaShield mounting medium with DAPI (Vector Laboratories, H-1200), and imaged on a DeltaVision Core microscope (Applied Precision Inc., Issaquah (WA), USA) as above.

### Macrophage infections

Peritoneal macrophages were extracted from CD1 mice and adhered overnight in RPMI medium (Sigma-Aldrich, R8758) with 10% heat-inactivated fetal calf serum (Gibco, 10500-064) onto 8-chamber tissue culture slides (Thermo Nunc Lab-Tek, 155409) at 37°C in 5% CO_2_. Macrophages were then infected with peanut lectin-purified metacyclic promastigotes at a ratio of 10 parasites per macrophage and imaged 18 or 48 h after infection in the DeltaVision Core environmental chamber at 37°C and 5% CO_2_, after DAPI staining (Molecular Probes, D1306) and 3 washes in PBS.

### Transmission electron microscopy

To monitor the ultrastructural features of autophagy in *L. major*, promastigotes in log phase of growth were washed 3 times in PBS and then starved by incubation in PBS for 4 h. Following starvation, promastigotes were sedimented and resuspended in a fixative solution containing 2.5% glutaraldehyde in cacodylate buffer 0.1 M, pH 7.2, for 1 h. After fixation, cells were washed twice in 0.1 M cacodylate buffer pH 7.2 and post-fixed in a solution containing 1% osmium tetroxide, 1.25% potassium ferrocyanide, and 5 mM calcium chloride in 0.1 M cacodylate buffer for 30 min at room temperature in the dark. The cells were then washed in the same solution before being dehydrated in increasing concentrations of acetone (30%, 50%, 70%, 90%, and 100%), and embedded in Epon. Ultrathin sections were obtained with a Leica ultramicrotome (Leica EM UC7, Wetzlar, Germany), stained with uranyl acetate and lead citrate and observed in a Tecnai G2 transmission electron microscope (FEI, Eindhoven, Netherlands) operating at 200 kV. The images were recorded using 4 k × 4 k CCD Camera (Eagle, FEI, Eindhoven, Netherlands). Parasites in stationary phase without starvation were also similarly processed and observed.

### Labeling of organelles

*L. major* promastigotes were incubated with 40 μM FM 4-64 (from 12 mM stock in DMSO; Molecular Probes, T-3166) at 4°C for 15 min then washed in PBS and resuspended in fresh medium before incubation at 25°C for 1 h to label the lysosomal compartment. Cells were then washed twice in PBS and processed for live cell microscopy. To induce mitochondrial fragmentation, log phase parasites were incubated in medium with 1 mM H_2_O_2_ for 1 h at 25°C, before washing in PBS and preparing for live microscopy as above.

### Statistical methods

Statistical analyses were performed using GraphPad Prism 5 software (GraphPad Software, La Jolla (CA), USA). Data were considered to be significantly different if the *P* value was less than 0.05.
